# LINE-1 ORF2p expression is nearly imperceptible in human cancers

**DOI:** 10.1186/s13100-019-0191-2

**Published:** 2019-12-31

**Authors:** Daniel Ardeljan, Xuya Wang, Mehrnoosh Oghbaie, Martin S. Taylor, David Husband, Vikram Deshpande, Jared P. Steranka, Mikhail Gorbounov, Wan Rou Yang, Brandon Sie, H. Benjamin Larman, Hua Jiang, Kelly R. Molloy, Ilya Altukhov, Zhi Li, Wilson McKerrow, David Fenyö, Kathleen H. Burns, John LaCava

**Affiliations:** 1 McKusick Nathans Department of Genetic Medicine, Johns Hopkins University School of Medicine, Baltimore, MD 21205 USA; 2 Department of Pathology, Johns Hopkins University School of Medicine, Baltimore, MD 21205 USA; 3 Institute for Systems Genetics, Department of Biochemistry and Molecular Pharmacology, NYU School of Medicine, New York, NY 10016 USA; 4 Laboratory of Cellular and Structural Biology, The Rockefeller University, New York, NY 10065 USA; 5 Department of Pathology, Massachusetts General Hospital, Harvard Medical School, Boston, MA 02114 USA; 6 Laboratory of Mass Spectrometry and Gaseous Ion Chemistry, The Rockefeller University, New York, NY 10065 USA; 7 Moscow Institute of Physics and Technology, Dolgoprudny, 141701 Russia; 8 European Research Institute for the Biology of Ageing, University Medical Center Groningen, Groningen, 9713 AV The Netherlands

## Abstract

**Background:**

Long interspersed element-1 (LINE-1, L1) is the major driver of mobile DNA activity in modern humans. When expressed, LINE-1 loci produce bicistronic transcripts encoding two proteins essential for retrotransposition, ORF1p and ORF2p. Many types of human cancers are characterized by L1 promoter hypomethylation, L1 transcription, L1 ORF1p protein expression, and somatic L1 retrotransposition. ORF2p encodes the endonuclease and reverse transcriptase activities required for L1 retrotransposition. Its expression is poorly characterized in human tissues and cell lines.

**Results:**

We report mass spectrometry-based tumor proteome profiling studies wherein ORF2p eludes detection. To test whether ORF2p could be detected with specific reagents, we developed and validated five rabbit monoclonal antibodies with immunoreactivity for specific epitopes on the protein. These reagents readily detect ectopic ORF2p expressed from bicistronic L1 constructs. However, endogenous ORF2p is not detected in human tumor samples or cell lines by western blot, immunoprecipitation, or immunohistochemistry despite high levels of ORF1p expression. Moreover, we report endogenous ORF1p-associated interactomes, affinity isolated from colorectal cancers, wherein we similarly fail to detect ORF2p. These samples include primary tumors harboring hundreds of somatically acquired L1 insertions. The new data are available via ProteomeXchange with identifier PXD013743.

**Conclusions:**

Although somatic retrotransposition provides unequivocal genetic evidence for the expression of ORF2p in human cancers, we are unable to directly measure its presence using several standard methods. Experimental systems have previously indicated an unequal stoichiometry between ORF1p and ORF2p, but in vivo, the expression of these two proteins may be more strikingly uncoupled. These findings are consistent with observations that ORF2p is not tolerable for cell growth.

## Background

Mobile elements make up nearly half of the human genome [1, 2]. The most prevalent sequences are retrotransposons, which propagate via RNA intermediates, and of these, modern activity resides with the Long INterspersed Element-1 (LINE-1, L1) sequences and those elements mobilized by L1 proteins in *trans* (reviewed in [3–5]). L1 is the only autonomous (protein-coding), functional retrotransposon in humans, and each of us inherits a distinct complement of active elements [6]. Mobilization occurs after a retrotransposition-competent L1 is transcribed, translated into proteins encoded by its open reading frames (ORFs), ORF1p [7, 8] and ORF2p, and packaged into a ribonucleoprotein (RNP) complex [9, 10]. ORF2p encodes an endonuclease [11] that cuts the genomic DNA target site and a reverse transcriptase [12] that generates L1 cDNA.

Many malignant tissues undergo L1 promoter hypomethylation [13–17] and permit L1 expression and somatic retrotransposition [13, 14, 18–26]. Both targeted and genome-wide sequencing efforts have identified thousands of de novo insertions that have occurred across hundreds of human cancers. Several groups have shown that L1 ORF1p expression is a hallmark of many different cancers [23, 27]. Of these many cancer types, it has been shown that L1 upregulation is induced early in the development of ovarian cancers, where ORF1p accumulation is evident within precursor lesions of the fallopian tube [15, 28]. L1 retrotransposition can also contribute directly to cellular transformation; in colon cancers, acquired L1 insertions are known to cause driving mutations in the adenomatous polyposis coli (*APC*) tumor suppressor [13, 29].

ORF2p is strictly required for retrotransposition [30], and so its expression in human malignancies can be inferred. Whether the protein can be directly detected has been a matter of some debate. In experimental systems, ORF2p is translated from the bicistronic transcript through an unconventional mechanism [31]. Compared to ORF1p, ectopically expressed ORF2p accumulates in substoichiometric amounts (ORF1p:ORF2p ratio > 30:1) and may be restricted to a subset of cells within a population [9, 10, 32]. Reports of endogenously expressed ORF2p have been more limited than ORF1p [33]. To our knowledge, to date, two groups have independently reported development of monoclonal antibodies recognizing human ORF2p, both using BALB/c mice. One reagent, developed by Belancio and colleagues [34], was reported to detect ectopically expressed ORF2p only. A second reagent, developed by Sciamanna, Spadafora, and colleagues [35], was reported to detect endogenous ORF2p in several malignant tissues where ORF1p expression has been reported. However, questions have been raised about the specificity of this reagent (Logan and colleagues). The difficulty directly detecting ORF2p may not simply be a matter of lacking exceptional affinity reagents for sensitive and specific western blotting. As we describe in this report, ORF2p has also eluded robust detection in a systematic, mass spectrometry-based tumor proteome sequencing effort (breast and ovary analyzed here) and in our own immunoprecipitations of ORF1p from resected patient colorectal tumors; this, when ORF1p is robustly detected and captured.

Here, we present our perspective on ORF2p detection, including results obtained searching for ORF2p in cancer proteomes as well as probing and analyzing tissue sections and immunoprecipitates. We describe the development of additional reagents to detect ORF2p: 5 rabbit monoclonal antibodies. Western blotting, immunoprecipitation, immunohistochemistry, and immunofluorescence demonstrate the utility of these reagents in experimental systems with ectopic LINE-1 expression. However, we have not yet detected endogenous ORF2p using these approaches; moreover, we report endogenous ORF1p-associated interactomes, affinity isolated from colorectal cancers (CRC), in which ORF2p was not found. These studies indicate that ORF2p is expressed at presently undetectable levels in human cancers, corroborating previous studies by Sokolowski et al.[34].

## Results

### Detecting ORF1p and ORF2p peptides in tumor mass spectrometry data

We reanalyzed data from Clinical Proteomics Tumor Analysis Consortium (CPTAC) to assess L1 ORF1p and ORF2p protein production in tumors. CPTAC has generated deep mass spectrometry based proteomics data from treatment naive breast [36] and ovarian [37] tumors using isobaric labeling and extensive prefractionation with alkaline reversed-phase chromatography followed by inline (acidic) reversed-phase chromatography and Orbitrap mass spectrometry. For the detection of ORF1p and ORF2p peptides, we constructed a protein sequence collection that, in addition to human proteins from Ensembl, also included high confidence LINE-1 protein coding sequences from L1Base2 [38], and used the X! Tandem [39] search engine with the curated databases and the same search parameters as Ruggles et al. [40].

**Fig. 1:**
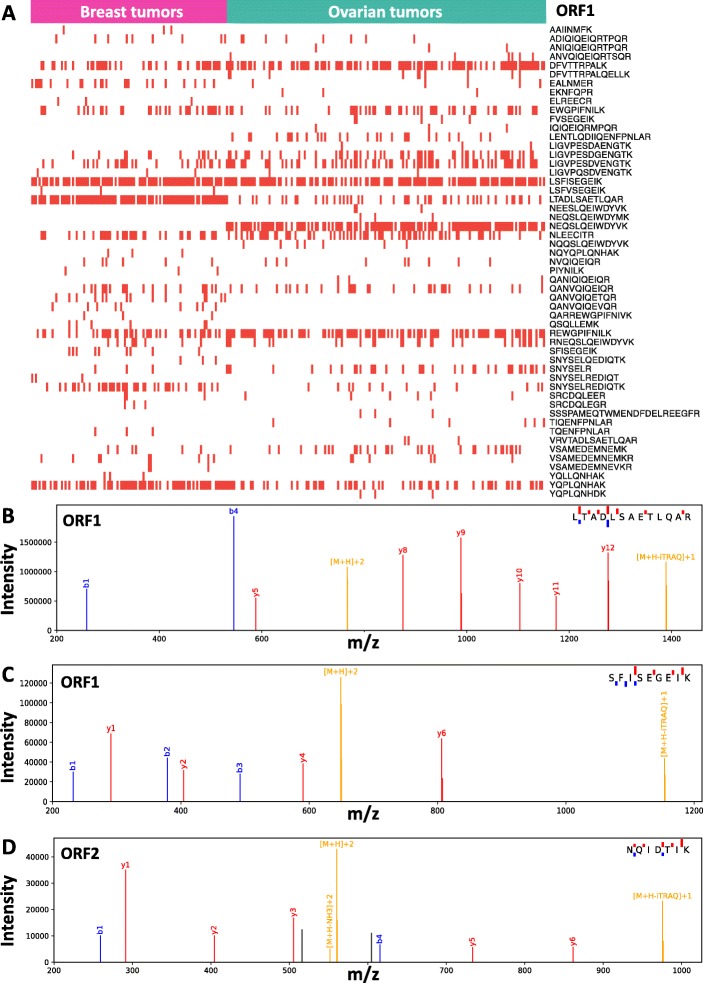
LINE-1 Peptide Detection in Tumor Mass Spectrometry Data. (**a**) ORF1p peptides observed in CPTAC breast and ovarian tumors. Each column represents a tumor-derived MS dataset (102 fuchsia-colored columns for breast tumors and 176 sea foam-colored columns for ovarian tumors) analyzed for the presence of L1 ORF peptides. ORF1 peptides, displayed at the right, mark rows. A red tick indicates that the given peptide was detected as present in the according tumor sample (white space: peptide not detected). Highest quality PSMs that were observed for (**b**, **c**) ORF1p and (**d**) ORF2p are displayed. Precursor ion related peaks are shown in yellow, y-ions in red, b-ions in blue, and unassigned ions in black

We observed ORF1p in most breast and ovarian tumors (Fig. 1a, with several peptides observed for the majority of tumors; see Fig. 1b and c for two examples of quality ORF1p peptide spectrum matches [PSMs]), but there was no clear evidence for ORF2p. Even when we relaxed the filters, the potential evidence for ORF2p peptides was questionable and the majority of PSMs were semi-tryptic and had borderline e-values (≈ 0.01). We also inspected the potential ORF2p PSMs manually and rejected them because they had several large peaks that could not be explained by fragmentation of the assigned ORF2p peptide. The best ORF2p PSM and only potential evidence for ORF2p that was not rejected is shown in Fig. 1d, but this peptide is short and still has two prominent peaks that are not explained by the sequence. In summary, we can reliably observe ORF1p in breast and ovarian tumors using deep mass spectrometry-based proteomics, but, in contrast, the evidence for detection of ORF2p is inconclusive.

### Monoclonal antibodies detect human LINE-1 ORF2 protein

**Fig. 2:**
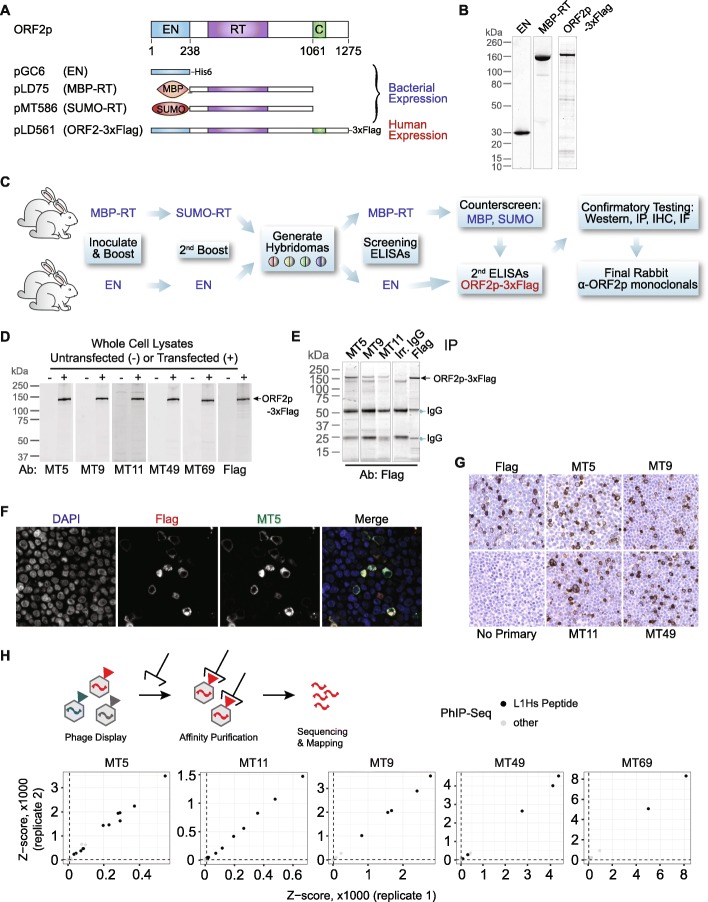
Production of monoclonal ORF2p antibodies. **a** Expression constructs used to generate antigens for ORF2p antibody production. **b** Coomassie-stained protein electrophoresis gels illustrating purity of ORF2p antigens used in antibody generation. **c** Immunization strategy to produce rabbit monoclonal antibodies. **d** Western blot detection of overexpressed ORF2p-3xFlag obtained from HEK-293T_LD_ cells transfected with pLD561 (shown in panel a) using 5 different monoclonal antibodies (Ab) compared to anti-Flag. **e** Immunoprecipitation of ORF2p-3xFlag using 3 antibodies. **f** Immunofluorescence imaging of HEK-293T_LD_ cells expressing ORF2p-3xFlag showing co-localization with anti-Flag antibody. **g** Immunohistochemistry of HEK-293T_LD_ cells expressing ORF2p-3xFlag with 4 monoclonal antibodies compared to anti-Flag. **h** Above, overview of PhIP-Seq. A phage library expresses protein epitopes from the protein-coding genome, which are affinity purified with ORF2p antibodies. DNA sequences are then isolated and sequenced to identify the genes encoding the peptides. Below, results from five monoclonal antibodies targeting ORF2p. In each instance, the greatest affinity of the ORF2p monoclonal antibodies is for peptides encoded by L1Hs ORF2p peptides. EN = endonuclease, RT = reverse transcriptase, MBP = mannose binding protein, SUMO = small ubiquitin-like modification

To pursue targeted ORF2p detection methods, we chose the retrotransposition-competent L1RP sequence as an immunogen for generating ORF2p monoclonal antibodies. L1RP is part of the highly active Ta-1d subfamily of L1, which encompasses the vast majority of hot L1 s found in humans (including LRE3, L1RP, L1.3) [41–43]. Prior to immunization in rabbits, we expressed tagged ORF2p fragments from bacteria, one fragment with the endonuclease domain (EN, amino acids 1–238, His6 tag) and one fragment containing the reverse transcriptase domain and surrounding sequence (RT, amino acids 238–1061, tagged with mannose binding protein/MBP or a small ubiquitin-like modifier/SUMO) (Fig. 2a). We also expressed full-length Flag-tagged ORF2p (ORF2-3xFlag) in Tet-On human embryonic kidney-293T (HEK-293T_LD_) cells to screen immune sera. We confirmed fragment purity after Nickel or size-exclusion chromatography (Fig. 2b).

For EN-targeting antibodies, we immunized and boosted two rabbits with EN-His6 fragments, then screened hybridoma supernatants by ELISA against purified EN domain and subsequently ORF2-3xFlag. We used the same strategy for RT-targeting antibodies, but used MBP-RT to stimulate the primary immune response and boosted with SUMO-RT to avoid MBP-specific antibody generation. We counter-screened against MBP and SUMO immunoreactivity to identify hybridomas with reactivity for ORF2p (Fig. 2c). Hybridoma supernatants were then tested for their ability to detect ORF2-3xFlag by western blot (Fig. 2d), IP (Fig. 2e), immunofluorescence (IF, Fig. 2f), and immunohistochemistry (IHC, Fig. 2g). We used Flag-antibody as a control to determine whether our ORF2p antibodies detected ORF2-3xFlag. Five monoclonal antibodies (mAbs) were selected based on their ability to detect full-length ORF2-3xFlag by each modality: MT5, MT9, MT11, MT49, and MT69.

### ORF2p antibodies are specific for human-specific LINE-1 (L1Hs)

To assess the specificity of antibodies for L1Hs, we employed Phage-ImmunoPrecipitation Sequencing (PhIP-Seq) [44–46]. We obtained all annotated repeats in the human genome from RepeatMasker and constructed a representational phage-display library which was used in combination with a previously constructed pan human proteome library [47]. The RepeatMasker peptidome was tiled from N- to C-terminus using 56 amino acid peptides with 28 amino acid overlaps. PhIP-Seq enabled us to determine both on- and off-target reactivities of each antibody by sequencing library IPs (Fig. 2h). Each of the five mAbs pulled down several peptides encoded by L1Hs sequences with high significance across both replicates. IP of L1Hs-derived peptides was orders of magnitude more significant compared to any other peptide encoded by the unique and repeat human genome included in the phage libraries. Based on these data, we conclude that these mAbs have minimal off-target reactivity, suggesting that these mAbs should be highly specific to the L1Hs ORF2p.

### ORF2p antibodies identify non-overlapping epitopes

There are several hundred potentially active L1 loci with intact open reading frames that have been characterized in modern humans [6, 48], and these sequences are highly identical to one another at both the nucleotide and amino acid level. The specific repertoire of L1 loci that is expressed varies among individuals, and by cell or tissue type [13, 14, 49, 50]. To evaluate the potential of our mAbs to detect proteins originating from distinct copies of L1Hs, we mapped the specific epitopes recognized by each antibody and evaluated the conservation of each epitope among L1Hs loci.

**Fig. 3:**
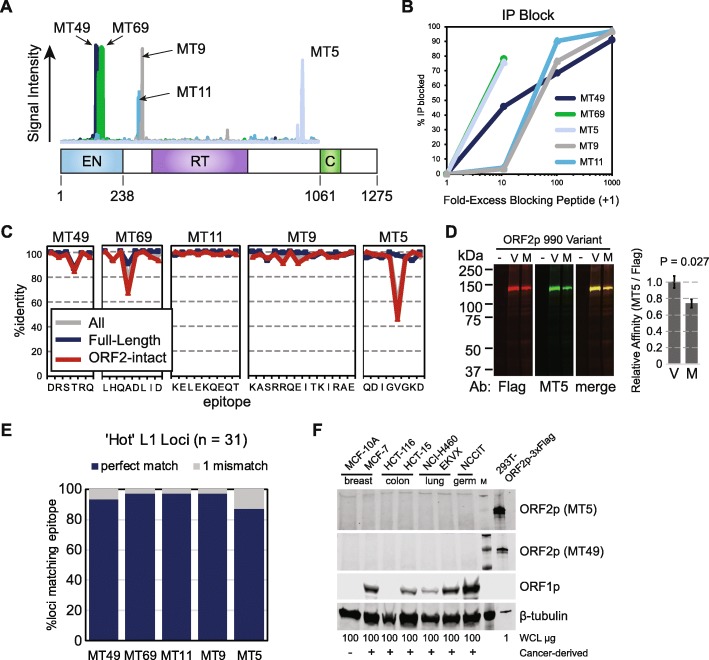
ORF2p mAbs detect endogenous L1Hs. **a** Epitopes identified by five ORF2p mAbs are indicated along the linear sequence of ORF2p. **b** IP blockade of ORF2p pulldown can be achieved by pre-incubating ORF2p mAbs with blocking peptides identified in (**a**). **c** ORF2p mAb epitopes are highly conserved among both full-length and ORF2-intact L1Hs sequences in the human genome. **d** Western blot measuring the ability of the MT5 antibody to detect an L1Hs polymorphism at amino acid position 990 reveals that the antibody can detect both alleles. **e** Epitope %identities among 31 ‘hot’ or highly active L1Hs sequences as reported by Brouha et al. [49]. **f** Western blot of whole cell lysates (WCL) from several ORF1p negative and ORF1p positive cancer cell lines fails to detect ORF2p with two different ORF2p mAbs. HEK-293T_LD_ cells expressing ORF2-3xFlag are included as a positive control

We mapped target epitopes using a peptide array of overlapping 15-mers tiling the length of ORF2p. We incubated these arrays with each antibody and then used secondary antibodies conjugated to horseradish peroxidase to identify which peptides were identified. Epitopes were identified as the largest contiguous stretch of amino acids that showed mAb binding over background (Fig. 3a). The linear epitopes ranged in length from 6 to 14 amino acids. Each of the 5 epitopes mapped to a discrete, non-overlapping segment of ORF2p in a manner consistent with the purified protein fragments we used for rabbit immunization. The MT49 epitope (DRSTRQ) and MT69 epitope (LHQADLID) occur adjacent to one another and target amino acids on the surface of the endonuclease domain according to a published crystal structure [51]. Both the MT9 epitope (KASRRQEITKIRAE) and MT11 epitope (KELEKQEQT) are located between the annotated EN and RT domains, whereas MT5 identifies an epitope (QDIGVGKD) ~300 amino acids from the C terminus, adjacent to the C domain.

To validate these epitopes, we pre-incubated mAbs with blocking peptides and attempted IP of ORF2-3xFlag. We found a concentration-dependent blocking activity of each peptide on its corresponding mAb; a range of 10–1000-fold excess peptide was required to achieve this effect depending on the mAb (Fig. 3b). These findings confirm that these epitopes are the antibody targets. PhIP-Seq data were also consistent with these being the cognate epitopes recognized by each antibody. Finally, we complemented our finding of antibody specificity by PhIP-Seq by performing a BLAST search of these epitopes, which revealed that the only perfect matches in the human genome belong to L1 ORF2p sequences.

### ORF2p mAbs are sensitive for many genomic source elements

To evaluate the occurrence of these epitopes in naturally-occurring L1 sequences, we used a census of fixed and commonly-occurring potentially protein-coding L1 elements found in the hg38 reference genome build. We focused on those with intact ORF2 reading frames as previously annotated by L1Base [38, 52]. We performed clustal alignments for two non-overlapping sets of these elements, one consisting of 146 full-length loci (111 L1Hs, 35 L1PA2) and one with 107 ORF2-intact loci. We included consensus sequences of the youngest human-specific L1 (L1Hs) and next-youngest primate-specific L1 (L1PA2) as well as the sequence of L1RP – the antigen against which our mAbs were raised – to compare sequences of the immunogen used for antibody generation against those of other genomic L1 loci. Full-length, intact LINEs are predominantly of the species-specific L1Hs subfamily, but include some older, primate-specific L1 elements. As expected, full-length and ORF2-intact L1 amino acid sequences are nearly identical for this set (Additional file 1: Figure S1). L1RP-encoded ORF2p is 1275 amino acids long. Individual, full-length L1 elements had open reading frames that differed from this on average by 16 amino acid variants (1.25%, range 1–61, Additional file 1: Figure S1), and ORF2-intact L1 loci differed on average by 32 amino acid variants (2.5%, range 2–79, Additional file 1: Figure S1).

To assess which of the several hundred reference L1Hs loci could be detected by our mAbs, we used these clustal alignments to evaluate the proportion of L1 loci matching each mAb epitope (Fig. 3c). For each epitope, most full-length L1 loci have amino acid sequences that are 100% identical. The MT11 epitope (KELEKQEQT) and MT9 epitope (KASRRQEITKIRAE) similarly occur nearly universally in ORF2-intact L1 sequences. The greatest discrepancy occurred in the MT5 epitope (QDIGVGKD), where amino acid position 990, which tends to be universal in intact, full-length L1 sequences, is not consistently found in elements selected only for an intact ORF2. Position 990 is typically a valine in L1Hs sequences and a methionine in older elements such as L1PA2 due to a G > A nucleotide substitution (ORF2 position 2968). We tested whether substituting the L1Hs valine for methionine was sufficient to preclude antibody recognition of the epitope. We created Flag-tagged L1RP ORF2p with an M990 substitution, expressed both protein variants in HEK-293T_LD_, and performed a western blot using both MT5 and anti-Flag antibodies (Fig. 3d). We detected no signal from MT5 or anti-Flag in untransfected cells. We detected both V990 and M990 variants with both anti-Flag and MT5 antibodies. The M990 variant was detected at a weaker relative intensity by MT5 compared to anti-Flag, indicating a reduced affinity for the ancestral (M990) sequence compared to the derived (V990) sequence. Consequently, we concluded that this single amino acid change within the target epitope may reduce detection sensitivity but would not prevent detection of L1PA2-encoded ORF2p by this reagent. Among 31 loci previously reported to be ‘hot’ or highly active elements [48], mAb epitopes differ by at most 1 amino acid from the L1RP variant (Fig. 3e, and Additional file 2: Table S1), suggesting that these youngest L1Hs sequences are likely identifiable by all of the 5 antibodies. It is important to note that these reference L1 sequences do not capture all of the sequence variation within the many polymorphic L1 alleles that are currently segregating in human populations; these are likely close in sequence to the L1Hs consensus but will differ by some number of amino acids. Thus, without having actual sequence data, our reagent may fail to bind some variants. However, we expect that our mAbs can detect the large majority of active L1 encoded in the human genome. We thus probed ORF2p expression by western blot in a panel of cancer cell lines known to express ORF1p but were unable to detect it (Fig. 3f).

### Characterizing L1 immunoprecipitates from CRCs

**Fig. 4:**
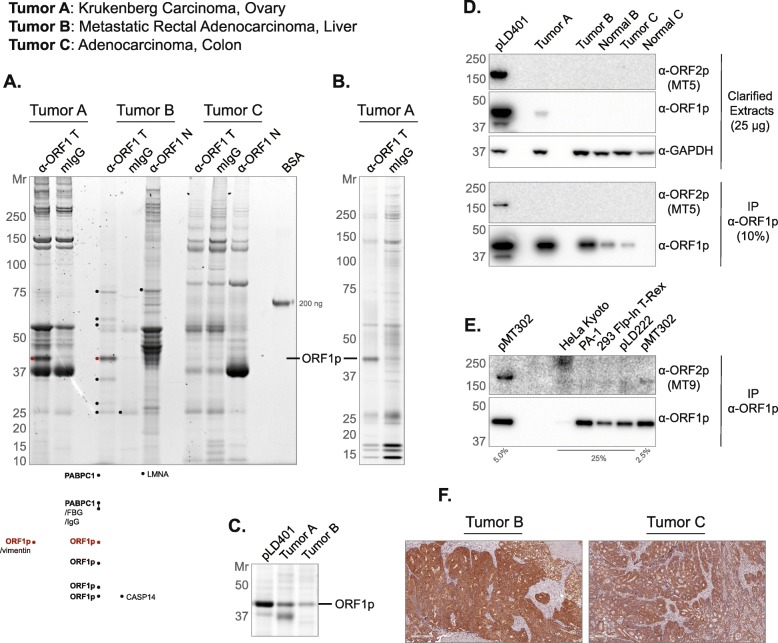
Protein Staining and Western Blotting of anti-ORF1p IPs and extracts. **a** Three tumors (labeled TOP, LEFT) were used as starting material for ORF1p affinity isolations (α-ORF1p T), including mock-capture controls using mouse IgG affinity medium with tumor extracts (mIgG), and matched normal tissue with anti-ORF1p affinity medium (α-ORF1p N). The eluted material was electrophoresed (4–12% Bis-Tris NuPAGE) and Coomassie G-250 stained [53]; a 200 ng BSA standard is displayed as a staining intensity gauge. Each lane contains a 200 mg-scale isolation using 10 μl of affinity medium. Several bands were cut and analyzed by LC-MS/MS - the highest-ranking proteins are listed (see Methods) (**b**) Tumor A anti-ORF1p affinity capture was repeated using a slightly modified procedure (see Methods). 30% of 100 mg-scale affinity isolations using 15 μl of affinity medium have been electrophoresed and Sypro Ruby stained. **c** Comparison of ORF1p yield from anti-ORF1p affinity isolations. pLD401 is a codon-optimized L1 sequence (*Orfeus*Hs), ectopically expressed in HEK-293T_LD_ [10]. Here, 80% of 100 mg-scale affinity isolations using 10 μl of affinity medium have been electrophoresed and Coomassie G-250 stained. **d** Western blotting of the same materials used in (**c**), including Tumor C and matched normal tissues. Here, 25 μg of the whole cell extract have been probed for ORF2p, ORF1p, and GAPDH as a control. 10% of α-ORF1p affinity isolates have also been probed for ORF2p and ORF1p. **e** A collection of cell lines were assessed by anti-ORF1p affinity capture. pMT302 is derived from a naturally occurring L1 sequence (L1RP), ectopically expressed in HEK-293T_LD_ [10]. pLD222 is a plasmid harboring a doxycycline-inducible GFP construct ectopically expressed in HEK-293T_LD_; here included as a control for pMT302. **f** IHC using α-ORF1p on (LEFT) Tumor B and (RIGHT) Tumor C. α-ORF2p clones MT5 (panel D) and MT9 (panel E) are described in this study (see Figs. 2 and 3)

We next pursued ORF2p detection after first affinity enriching ORF1p from tumor extracts. In our prior work with ectopically expressed L1 RNPs in HEK-293T_LD_ cells, we readily co-immunoprecipitated ORF1p/ORF2p/L1 RNA-containing macromolecules, and robustly detected ORF proteins [9, 10]. The detection of ORF2p had, at the time, been a widely recognized problem [33] which we addressed by appending a 3xFlag epitope-tag to the protein. The 3xFlag tag allowed us to robustly capture and detect ORF2p. Because these experiments provided a window on L1 biology only in an ectopic expression context, we wanted to evaluate concordance with pathophysiology. To this end, we sought to isolate L1 RNPs, directly from ORF1p-expressing tumors using an anti-ORF1p affinity medium, comparing and contrasting the results obtained with those from our studies of ectopic L1 expression. We obtained a cohort of CRCs that were shown to be ORF1p positive (+) by IHC and carried out a preliminary proteomic characterization of three tumors selected from across the ORF1p + IHC staining spectrum. Figure 4 shows the results we obtained by multiple proteomic methods. Tumor A was the highest ORF1p-expressing case among this group. Tumor C was on the low-to-moderate-end of the expression spectrum and did not yield a distinct, visible ORF1p band after IP: Fig. 4a, compare the ORF1p staining intensity in the first (far left, Tumor A), fourth (Tumor B), and eighth (Tumor C) lanes of the gel; Fig. 4b exhibits results obtained with Tumor A using a modified procedure (see Fig. 4 legend and Methods). Also, see Fig. 4c for a comparison of ORF1p yield after IP from our highest-expressing ectopic system (pLD401 [10]) to tumors A and B. Although IP from these materials yields ORF1p quantities that are directly comparable, side-by-side western blotting of cell extracts revealed that ectopic expression produced a significantly higher level of ORF1p than these tumors (Figs. 4d ). Surprisingly and importantly, ORF2p is only detected on our western blots in the ectopic expression positive control (Fig. 4d, e, and Additional file 3: Figure S4). The result did not change when extending the blot exposure time from 2 min (Fig. 4d, shown) to 30 min (not shown); nor was the result different when using alternative anti-ORF2p antibody clones characterized in this study. Presuming ORF2p has been retrieved from these tumors by co-IP with ORF1p, we conclude the yield is below the lower limit of detection of our blotting, under the conditions tested. Figure 4f shows cognate anti-ORF1p IHC staining results for tumors B and C – corroborating the signal intensity difference revealed by western blotting.

To contextualize the results obtained with CRCs, we developed a similar analysis in a broader selection of cell lines (Fig. 4e). We observed that the yield of endogenous ORF1p by IP was ≲ 1/10th the amount observed in HEK-293T_LD_ expressing L1 ectopically from pMT302. This construct was chosen on account of its milder ectopic expression level. Modified from a naturally occurring L1 sequence (L1RP), expression from pMT302 has been estimated to yield ~ 1/40th the L1 RNA and ORF2p and ~ 1/4th the ORF1p expression typically observed from codon-optimized L1 encoded by pLD401 [10, 33]. PA-1, an ovarian teratocarcinoma cell line known to be permissive for the expression of endogenous L1 [54], stood out among this group - demonstrating ~ 1/10th the ORF1p yield of pMT302 and the highest yield from an endogenous context in this panel. Western blotting also demonstrated ORF1p signal in cell lysates from the panel, but only under probing conditions that increased high mass (nonspecific) signal in the blot (Additional file 4: Figure S2). In this panel, ORF2p was not detected, except by co-IP with ORF1p from pMT302.

**Fig. 5:**
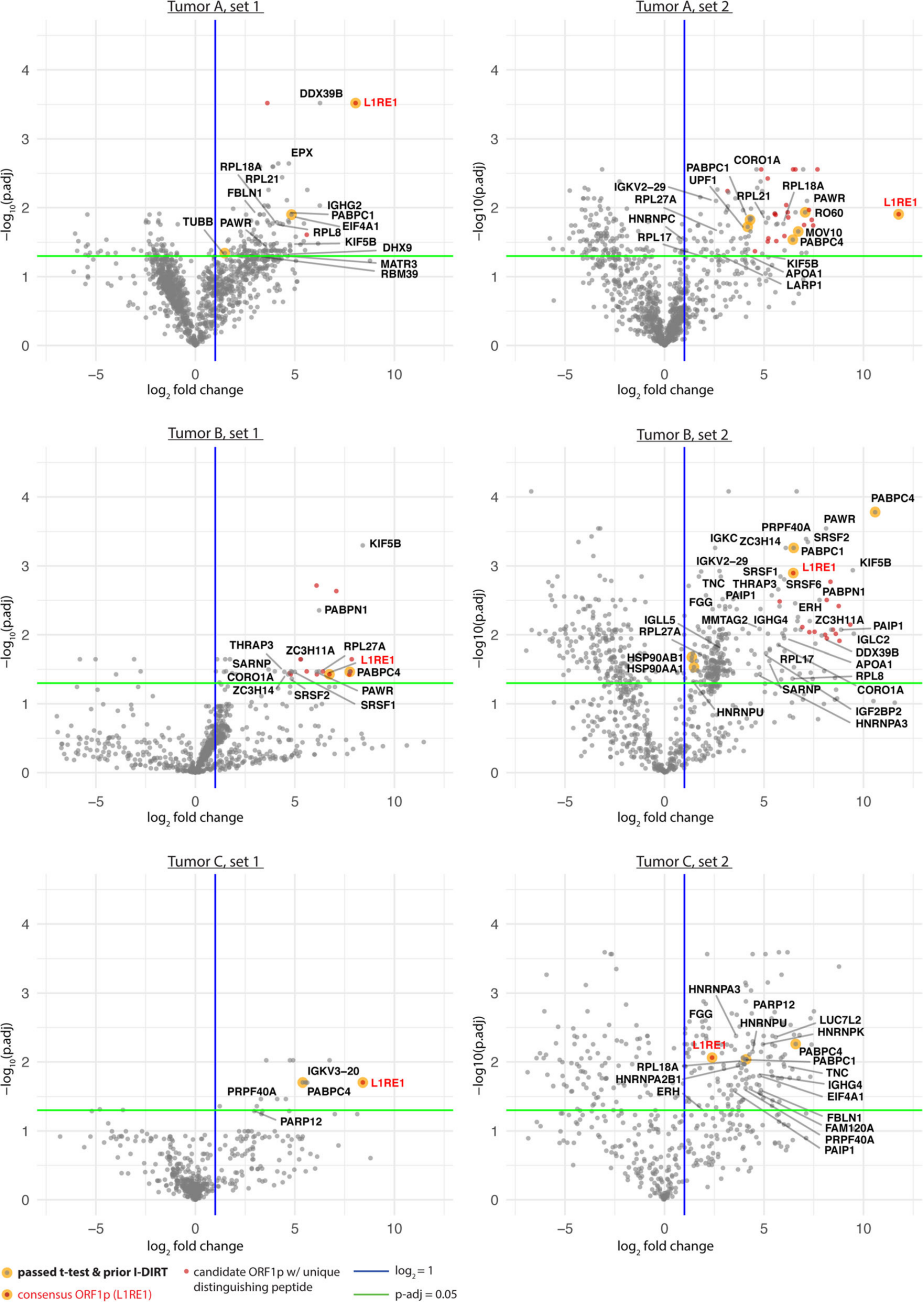
Label-free Quantitative IP-MS analysis. A legend appears at the bottom: attention is drawn to hits observed previously by I-DIRT, matches to L1RE1 (Uniprot: ORF1p consensus), and candidate non-consensus ORF1p sequences. Gene symbols corresponding to tumor-specific, quantified proteins are displayed on each plot with the following criteria: 1. the protein exhibited statistical significance in the IP (see **Methods**) with a log_2_ fold change ≥2 and also exhibited statistical significance in another IP from this study with log_2_ fold change ≥1; or 2. the protein was previously determined specific by I-DIRT or was highlighted in other literature (discussed in the main text) and exhibited statistical significance. **a** Tumor A, these IPs (set 1 and 2) differ in several experimental parameters (see Methods); both sets use a mock IP control (mouse IgG). **b** Tumor B, two distinct controls were used: (LEFT) mIgG IP, (RIGHT) matched normal liver, α-ORF1p IP. **c** Tumor C, controls as for Tumor B with matched normal colon. These data correspond to the IPs displayed in Additional file 5: Figure S3 and the results are collated in Additional file 6: Table S2

Believing we exhausted the potential of western blotting for ORF2p detection, we turned to MS-based proteomic analyses. Figure 5 displays the results of a label-free, quantitative MS analysis of affinity captured ORF1p, from the same tumor samples displayed and analyzed in Fig. 4 (see also Additional file 5: Figure S3). As expected, we identified L1RE1 (consensus ORF1p) as a significantly enriched protein in each IP set. Taken all together, we observed eight other proteins that we have previously characterized as putative physiological L1 interactors (PABPC1, PABPC4, TUBB, RO60, UPF1, MOV10, HSP90AA1, HSP90AB1); PABPC1/4 being most frequently recovered. We explored the interactors discussed in [55], originating from two studies, conducted by the Moran and Kazazian labs [56, 57]. We observed DHX9 and MATR3 (in Tumor A, set 1), HNRNPC and LARP1 (in Tumor A, set 2), SRSF1 (in Tumor B, set 1 & Tumor B, set 2), SRSF6 and IGF2BP2 (Tumor B, set 2), HNRNPU (in Tumor B, set 2 & Tumor C, set 2), and FAM120A and HNRNPA2B1 (in Tumor C, set 2). Only HNRNPU was observed to be a significant hit in two different patient tumors. Notably, HNRNPU, DHX9, MATR3, HNRNPC, and other RNA binding proteins have been reported to accumulate on L1 and retro-element-derived RNAs; in one hypothesis, insulating these sequences from nuclear RNA processing pathways that might otherwise be deleterious to the retro-element and host genes harboring these sequences [58, 59]. The data can be summarized as follows: 291 proteins were detected as significant in one comparison (tumor vs. control IP: p-adjusted value of ≤0.05 and log_2_ fold change >1), 37 passed two comparisons, and 22 passed three comparisons; 21 ORF1p candidates with one or more mutations from consensus ORF1p were detected and of these 12 were observed in both tumor A and tumor B. We observed a candidate phosphorylation site at S18 (156 PSMs from this study). The next most frequent candidate phosphorylation site, S27, received only 31 PSMs; both S18 and S27 phospho-sites have previously been reported [60, 61] and have been implicated as (1) functionally important for retrotransposition and (2) mediating an interaction with the peptidyl-prolyl cis/trans isomerase PIN1. The above described findings are further annotated and summarized in Additional file 6: Table S2. Importantly, we did not detect ORF2p in any of these tumor analyses.

The above proteomic analyses were conducted under the assumption that these tumors will harbor somatic retrotransposition events based on what has been reported in the literature [13–17]. Nevertheless, we could not rule out the possibility that these tumors did not express ORF2p. We therefore selected a tumor (tumor D: a sigmoid colon cancer that metastasized to liver) and carried out transposon insertion profiling by sequencing (TIP-seq) [19, 22, 62] to map new insertions and establish ORF2p activity. TIP-seq analysis revealed somatically acquired insertions in the primary tumor and its metastatic sites (data not shown). On this basis we conducted co-IP/western blot analysis on the same material in an effort to detect ORF2p (Additional file 3: Figure S4). No ORF2p was detected, verifying that even when new insertions are observed to occur, and when ORF1p has first been highly enriched, ORF2p detection remains challenging.

### Endogenous ORF2p expression cannot presently be directly detected in human cancers

In summary, given the lack of ORF2p detection by mass spectrometry of tumor extracts (Fig. 1), we tested for endogenous ORF2p expression in human cancers tissues and cell lines by western blotting, IP-western, IP-MS, and IHC (Figs. 3, 4 and 5). Western blotting and IP-western in several widely used human cell lines expressing endogenous ORF1p showed no evidence of ORF2p expression (Fig. 3f and Fig. 4e). We also conducted IHC in human CRCs with these reagents, including cases known to sustain somatic retrotransposition. ORF1p was readily detectable in all cases evaluated. ORF2p immunostaining, by contrast, showed no consistent signal over isotype controls under standard conditions. Under conditions employing a highly sensitive protocol, immunoreactivity over the isotype control was apparent only inconsistently as cytoplasmic staining with one of the antibodies (MT49, data not shown).

## Discussion

We know that many types of human cancers accumulate somatically acquired L1 insertions [13, 15, 17–22, 24–26, 63, 64]. These insertions have several sequence features that specifically indicate retrotransposition by ORF2p; therefore, we have a high degree of confidence that ORF2p is expressed along with ORF1p in these malignancies. As expected based on the expression level, we reliably detect ORF1p and report co-enriching ORF1p interactors from CRC samples. Many are proteins that we and others have previously validated. Among them, we noted the presence of nuclear RNA binding proteins (e.g. DHX9, HNRNPU, HNRNPC, MATR3) that have been reported by others as L1 interacting proteins. The significance of these in our ORF1p co-IPs is still debatable, particularly given data from our group and others that ORF1p has limited role and lifespan in the nucleus in cultured cells [9, 32]: do these represent co-assembly of ORF1p with nuclear RNAs (L1 or otherwise); are our ORF1p co-IP populations sampled from cytoplasmic pools of heterogeneous RNAs harboring these proteins [59]; or are these proteins binding ORF1p-containing macromolecules post-lysis (and are therefore artifacts of spurious binding to L1 RNPs in vitro)? Standard LFQ MS control samples, including those used here, are not suited to rule out the latter scenario. Hence, more data are needed in order to dissect their potential significance to L1 molecular physiology.

In contrast to ORF1p, we have demonstrated that endogenous ORF2p expression is difficult to reliably directly detect in cancer samples despite strong genetic evidence for ORF2p enzymatic activity. This is true in the context of: (i.) highly fractionated tumor extracts subjected to mass spectrometry-based shotgun proteomic analysis (as part of the CPTAC initiative); (ii.) by employing high quality antibodies against ORF2p in standard western blotting, immunoprecipitation, and immunostaining protocols; and (iii.) anti-ORF1p affinity enrichment followed by western blotting and mass spectrometry. We also tried to IP ORF2p from CRC tissues and PA-1 cells using anti-ORF2p antibodies, followed by western blotting against ORF2p, but were unable to detect ORF2p (not shown); further optimizations may alter this outcome. While preparing this manuscript, a dataset was released comprising anti-ORF1p co-IPs from H9 human embryonic stem cells with LFQ MS-based analysis [65], which have been shown to exhibit strong ORF1p expression [66]. ORF2p detection was not reported. ORF2p peptide detection has previously been reported by MALDI-TOF MS after IP with an anti-ORF2p polyclonal antibody [67]; however, this reagent no longer exists, so further validation and side-by-side comparisons are not possible.

From a bioinformatic perspective, the mass spectrometric evidence for ORF1p is strong, as several high-quality peptide spectrum matches are observed and their intensities are highly correlated. In contrast, only one ORF2p peptide was observed to have reasonable PSMs (Fig. 1d) but even these have two unexplained medium intensity peaks – increasing the uncertainty that it constitutes an observation of ORF2p. We are also hesitant to claim that a protein is observed based on a single relatively short peptide even if it only maps to ORF2p and not to any other protein in the reference human proteome, as it might map to a variant that is present in the sample and not in the reference. In summary, the mass spectrometric evidence for ORF2p in tumors is, at best, questionable. We therefore recommend that ORF2p searches of mass spectrometry data implement additional filters to reduce the likelihood of false positives – these may include the rejection of ORF2p peptide matches when:

1. they exhibit poor fragmentation, particularly where the sequence differs from consensus;
2. the sequence could be explained by deamidation of consensus sequence (D or E in variant corresponds to N or Q in consensus, respectively);
3. the sequence could be explained by a non-tryptic cleavage of consensus sequence; and
4. the peptide matched is not fully tryptic.

Filter 1 is not practical to carry out manually on large data sets, but computational approaches are possible. Filters 2, 3, and 4 should be relatively straight forward to execute programmatically in future studies. The difficulty in detecting ORF2p has not been missed by the field, yet the enthusiasm to address this challenge may have generated some false starts (we are aware of a newly submitted manuscript from Susan Logan and colleagues re-examining the reliability of the chA1-L1 antibody [35]). Although we cannot rule out the presence of detectable levels of ORF2p in tumors examined by others, failure to reliably, directly detect endogenous ORF2 protein in the present study most likely points to an extremely low steady state abundance for this protein - even in the conditions of L1 de-repression that typify these cancers - in keeping with historical estimates of low ORF2p copy number and high ORF1p:ORF2p stoichiometry [10, 31]. Commonly used ectopic expression systems may not just overexpress ORF1p and ORF2p, but may also skew their relative abundances and/or apparent stoichiometry in resulting macromolecules [10]. If this is so, whether these experimental systems change the efficiency of ORF2p translation or overwhelm cellular clearance pathways is unclear. In this study we presented two direct analyses of ORF1p/2p co-IP yields from tumors vs. ectopic expression in HEK-293T_LD_ cells. Firstly, consider Fig. 4d; compare ORF1p and ORF2p signals from pLD401 to tumor A. The lower absolute expression level and recovery of ORF1p from tumor A suggests that the concomitant ORF2p yield by co-IP may have been too low to expect reliable ORF2p detection (at least, using anti-ORF2p clone 5), even at the apparent stoichiometry described for ectopic expression in HEK-293T_LD_ [10]. Now consider Additional file 3: Figure S4: compare ORF1p and ORF2p signals from pMT302 to tumor D. In this case ORF1p quantities were calibrated such that the yields from tumor D and ectopic expression in HEK-293T_LD_ were in a comparable range. The result shows that, for tumor D, under these experimental conditions, the recovery of ORF2p during co-IP is relatively decreased - to below the limit of detection - compared to ectopic expression in HEK-293T_LD_. Although caveats may apply, a simple conclusion is as follows: either ORF2p from the tumor is less stably associated with ORF1p under the same conditions of capture or the intrinsic abundance of ORF2p is lower, resulting in fewer ORF2p-containing RNPs.

Moreover, whether there is heterogeneous expression of ORF2p in tumors has not been addressed. It is possible that relatively rare malignant cells accumulate detectable amounts of ORF2p, and that these have escaped sampling in our IHC experiments. Taylor et al. previously observed that, in the presence of robust ectopic ORF1p expression, only a subset of cells - approximately one-third - also exhibited ORF2p expression [10], indicating a potentially cell-dependent stochastic determinant for ORF2p expression. Similarly, although somatic retrotransposition events result in acquired genomic L1 insertions that are propagated by the clonal expansions of tumors, we do not know whether these accumulate continuously over time or whether instead they reflect discrete, episodic breaches of host defenses against L1. Indeed, there may be active selection against cells with high ORF2p because of cytotoxic effects of the protein or retrotransposition products [68, 69].

## Conclusions

Here, we have evaluated L1 ORF2p expression in human cancers using several independent and orthogonal approaches - one reliant on whole proteome analysis; one employing a series of new, apparently avid and specific monoclonal antibodies for ORF2p detection; and one leveraging ORF1p interactions to seek evidence of ORF2p. While many types of epithelial cancers express levels of ORF1p that are directly detectable by western blotting and mass spectrometry, ORF2p in these cases appears to be only indirectly detectable by gDNA sequencing of de novo L1 insertions. The apparent uncoupling of ORF1p and ORF2p expression is striking, and potentially much more pronounced in vivo than in previously characterized experimental systems. We expect that in the future, more sensitive assays will reveal the now imperceptible quantities of ORF2p. If shotgun proteomic approaches are not sufficient to address ORF2p detection, targeted methods can be developed to maximally leverage the sensitivity of MS instruments (potentially ~100 s of attomoles [70]). Similarly, approaches such as proximity ligation assays (PLA) or other technologies, may amplify detection by antibodies. Characterizing ORF2p expression and understanding its regulatory mechanisms may have translational importance. If high levels of ORF2p expression are not compatible with malignant cell growth, restoring expression of this protein selectively in cancer cells producing L1 RNA and ORF1p may provide an avenue for therapeutics.

## Methods

### Detection of L1 ORF peptides in CPTAC data

The CPTAC discovery breast [36] and ovarian [37] mass spectrometry data was used (available at the CPTAC Data Portal: https://cptac-data-portal.georgetown.edu/cptac/s/S015 and https://cptac-data-portal.georgetown.edu/cptac/s/S020, respectively). For the detection of ORF1p and ORF2p peptides, we constructed a protein sequence collection that, in addition to human proteins from Ensembl, also included high confidence LINE-1 proteins from L1Base2 [38]: 292 ORF1p/ORF2p sequences translated from full-length intact LINE-1 and 107 ORF2p translated from ORF2 intact LINE-1 elements in human, and 89 LINE-1 ORF1p/ORF2p translated from ancestor consensus sequences. In addition, we also included a list of contaminant proteins from the common Repository of Adventitious Proteins (cRAP). We used the X! Tandem [39] (https://www.thegpm.org/tandem/) search engine with the curated databases and the same search parameters as in [40]. In-house scripts were used to parse the X! Tandem outputs to filter for high-quality Peptide-Spectrum Matches (PSMs). Only PSMs that meet the following criteria were retained: the fraction of the intensity of peaks that matched the sequence >40%, the gaps in the fragmentation were not larger than 3 amino acids, the peptide length > =7 and the e-value <= 0.01. We also eliminated PSMs that match to more than one gene. In order to select a set of reliable peptides from ORF1p, we performed a pair-wise comparison of the peptide quantities only kept the peptides that formed a set that had a Spearman correlation of 0.6 with each other.

### Purification of ORF2 proteins

pGC6, expressing ORF2p endonuclease, is tagged with N-HIS6-TEV. We expressed overnight in bacteria at 16°C, then shifted temperature and induced with IPTG. We froze cell pellets then purified on a nickel column in standard conditions. We cleaved the tag with TEV protease overnight, then performed gel filtration to clean up the untagged protein. pLD75, expressing ORF2p reverse transcriptase tagged with His-MBP, was expressed similarly, purified on a nickel column, then with cation exchange (HiTrap SP FF; GE Healthcare Life Sciences). pLD561, expressing full-length ORF2p-3xFlag, was expressed as a 15 L culture in suspension HEK-293T_LD_. We lysed cells with a microfluidizer in 500 mM NaCl buffer with 1% (v/v) Triton X-100, then performed Flag IP using Dynabeads (Thermo Fisher Scientific) coupled to anti-Flag M2 (Millipore Sigma) followed by 3xFlag elution.

### Generation of monoclonal antibodies

Rabbit monoclonal antibodies were developed with Abcam (Cambridge, MA). For EN-targeting antibodies, rabbits were immunized and boosted with EN, screened by ELISA for EN affinity, and then hybridoma supernatants were tested against ORF2p-3xFlag by ELISA. For RT-targeting antibodies, rabbits were immunized with MBP-tagged RT, boosted with SUMO-tagged RT, then screened by ELISA with MBP-RT and counter-screened with MBP and SUMO to eliminate clones that were specific for MBP or SUMO.

### ORF2p induction in HEK-293T_LD_ cells

Plasmid DNA was miniprepped using the Zyppy Miniprep Plasmid DNA kit (Zymo, Irvine, CA) or PureLink HiPure Plasmid Midiprep Kit (Thermo Fisher, Waltham, MA). These were transfected into Tet-On HEK-293T_LD_ cells [10] by incubating 3 μg plasmid DNA with 9 μL Fugene HD (Promega, Madison, WI) in 100 μL Optimem for 15 min, then adding dropwise to 6-well plates containing 500,000 cells per well. 1 μg/ml doxycycline was added at the time of transfection and cells were then used for immunoprecipitation, immunofluorescence, immunohistochemistry, or western blot assays 24 h later.

### ORF1p and ORF2p immunohistochemistry

For cells, HEK-293T_LD_ cells expressing a plasmid encoding ORF2-3xFlag were admixed with untransfected HEK-293T_LD_ and pelleted, fixed in 10% formalin for 24 h, then processed into paraffin-embedded blocks. For human tissue samples, de-identified paraffin-embedded blocks were obtained from the Pathology Department at Massachusetts General Hospital. Formalin-fixed paraffin embedded tissues were sectioned at 5 μm onto glass slides, heated to 65 °C for 20 min, and then rehydrated by serial washes in xylene, ethanol (100% / 90% / 75%), and water. IHC was performed with the DAKO EnVision+ System-HRP kit (cat# K4006, Agilent, Santa Clara, CA). Antigen retrieval was performed using Target Retrieval Solution for 20 min at >90°C, then slides were blocked with peroxidase block and then 2% (w/v) BSA in PBS. Primary antibody incubation with ORF1p was performed at 1:5000 for 1 h at room temperature and with ORF2p MT49 overnight at 4 °C at a final concentration of 10 μg/ml, and secondary HRP mouse polymer secondaries were used to label primary antibody with chromogen upon DAB addition. Hematoxylin was used as a nuclear counterstain. Slides were then dehydrated in serial washes and coverslips were placed. Scoring was performed by a trained pathologist.

### Immunofluorescence (IF)

HEK-293T_LD_ cells expressing a plasmid encoding ORF2-3xFlag were admixed with untransfected HEK-293T_LD_ and pelleted, fixed in 10% formalin for 24 h, then processed into paraffin-embedded blocks. IF was performed on 5 μM sections. Slides were processed as for IHC but using AlexaFlour-conjugated secondary antibodies (anti-rabbit 488 and anti-mouse 555). Imaging was performed on a Zeiss Confocal Microscope.

### Antibody epitope mapping

A library of peptide based epitope mimics was synthesized using solid-phase Fmoc synthesis. An amino functionalized polypropylene support was obtained by grafting with a proprietary hydrophilic polymer formulation, followed by reaction with t-butyloxycarbonyl-hexamethylenediamine (BocHMDA) using dicyclohexylcarbodiimide (DCC) with N-hydroxybenzotriazole (HOBt) and subsequent cleavage of the Boc-groups using trifluoroacetic acid (TFA). Standard Fmoc-peptide synthesis was used to synthesize peptides on the amino-functionalized solid support by custom modified JANUS liquid handling stations (Perkin Elmer). The binding of antibody to each of the synthesized peptides was tested in a pepscan-based ELISA. The peptide arrays were incubated with primary antibody solution (overnight at 4 °C). After washing, the peptide arrays were incubated with a 1:1000 dilution of anti-rabbit IgG HRP conjugate (DAKO) for 1 h at 25 °C. After washing, the peroxidase substrate 2,2′-azino-di-3-ethylbenzthiazoline sulfonate (ABTS) and 20 μl/ml of 3% H_2_O_2_ were added. After 1 h, the color development was measured with a charge coupled device (CCD) - camera and an image processing system. Epitope targets were read as the largest contiguous stretch of amino acids shared by all peptides recognized by the primary antibodies.

### Peptide blocking experiments

Blocking peptides were chosen to span two extra amino acids and were N-terminally acetylated and C-terminally amidated. Peptides were resuspended in acetic acid or ammonium acetate depending on their charge characteristics. To pre-block antibodies, 10, 100, or 1000-times excess peptide by weight was incubated with primary antibody mixture on a rotating wheel at 4 °C overnight. The next day, pre-blocked or no-block antibodies were conjugated to protein G dynabeads for 15 min at room temperature, then ORF2p-3xFlag HEK-293T_LD_ lysate was added for 1 h at room temperature. The remaining protocol is the same as described above for immunoprecipitation.

### Phage immunoprecipitation and DNA sequencing

The PhIP-Seq assay was described previously [46]. Approximately 100 ng of each mAb was added to the combined T7 bacteriophage human peptidome library (unique genome and repetitive element sublibrary addition, 1 × 105 plaque forming units for each phage clone in each library) and incubated with rotation overnight at 4 °C in deep 96-well plates in 1 mL total volume of phosphate-buffered saline. Negative controls for data normalization included eight mock immunoprecipitation reactions on each plate. mAb-phage complexes were captured by magnetic beads (20 μL of protein A-coated and 20 μL of protein G-coated, catalog numbers 10002D and 10004D, Invitrogen, Carlsbad, CA) for 4 h at 4 °C with rotation and processed using the Agilent Bravo liquid handling system (Agilent Technologies, Santa Clara, CA). Beads were washed twice with 0.1% NP-40 in Tris-buffered saline (50 mM Tris-HCl with 150 mM NaCl, pH 7.5), resuspended in 20 μL of a Herculase II-containing PCR mix (catalog number 600679, Agilent Technologies), and ran for 20 PCR cycles followed by a second 20-cycle PCR using 2 uL of the initial PCR products to add barcodes and P5/P7 Illumina sequencing adapters. Pooled PCR products were sequenced using an Illumina HiSeq 2500 (Illumina, San Diego, CA) in rapid mode (50 cycles, single end reads). Data were normalized and analyzed using a z-scores algorithm according to Yuan et al. [71].

### Cloning to generate ORF2p M990 variant

A plasmid with full-length codon-optimized L1 (pMT491) was digested with NotI-AscI, blunted with T4 polynucleotide kinase, and ligated with T4 DNA ligase to generate a doxycycline-inducible ORF2-3xFlag expression vector (pDA033). To generate the M990 mutant (Plasmid pDA101), we performed a 3-fragment multichange isothermal assembly [72] using BsrGI/BstZ17I-digested pDA033 as the backbone (fragment 3), and PCR-amplified fragments containing the V990 M mutant in the overlapping sequence. PCR fragments were amplified from pDA033 with Q5 polymerase (NEB) and assembly was performed with the HiFi Assembly Master Mix (NEB). Individual clones were screened for M990 mutations by Sanger sequencing. Fragment 1 was generated with primers 5′-TGAGCGGCTACAAGATCAACGTG-3′ and 5′-GCTCATGAAGTCCTTGCCCATGCCGATGTCCTGGATGGTG-3′. Fragment 2 was generated with primers 5′-CACCATCCAGGACATCGGCATGGGCAAGGACTTCATGAGC-3′ and 5′-ACATGTGCACATTGTGCAGGT-3′.

### Immunoprecipitation

For Figs. 4, 5, Additional file 3: Figure S4: handling of cryomilled HEK-293T_LD_ cells ectopically expressing L1 from pLD401 and pMT302 was previously described [10, 73]. Patient samples were milled and extracted similarly, as previously described [74]. Protein extraction solution: 20 mM HEPES pH 7.4, 500 mM NaCl, 1% (v/v) Triton X-100, 1x Roche Complete EDTA-free protease inhibitors. Tumor A was extracted in a separate instance in the same solution with the addition of Promega recombinant RNasin at 1:50 (v:v).

For patient samples subjected to LFQ-MS we used the following parameters: 200 mg-scale, 10 μl of anti-ORF1p (Millipore Sigma #MABC1152) and mouse IgG (Millipore Sigma #I5381) affinity medium were used per 200 mg-scale affinity capture. In addition to the mouse IgG mock affinity capture control, for tumors B and C, we carried out an additional mock affinity capture using the anti-ORF1p antibody and extracts from matched normal tissue, resected at the time the CRC was removed from the patient. Affinity media and clarified extracts were incubated for 1 h at 4 °C, washed three times with extraction solution, and eluted with NuPage sample buffer (Thermo Fisher Scientific #NP0007) at 70 °C. After SDS-PAGE (Thermo Fisher Scientific: 1 mm, 4–12% Bis-Tris NuPAGE system), samples were analyzed by general protein staining, western blotting, and/or MS as described in the main text. Samples destined for MS were reduced (DTT) and alkylated (iodoacetamide) prior to electrophoresis. In a second instance, tumor A affinity isolations were conducted at a 100 mg-scale using 15 μl of anti-ORF1p and mouse IgG medium, were extracted and washed (3 × 250 μl washes as opposed to 1 ml) in the presence of 1:50 RNasin (not previously included), and 1x protease inhibitors (normally only present during extraction); approximately ^2^/_3_ the standard sonication energy was applied (the standard is 15–20 J per 100 mg-scale in a 25% (w:v) extract). In all cases, representative SDS-PAGE lanes are displayed in Fig. 4 and the gel plugs used for LFQ-MS are displayed in Additional file 5: Figure S3. Unless otherwise stated, all panels displayed have been ‘auto tone’ calibrated, respectively, in Adobe Photoshop to maximize the visual contrast across the detected signal range.

For Figs. 2 and 3: HEK-293T_LD_ cells transfected with a plasmid encoding ORF2-3xFlag were lysed by sonication in extraction buffer (50 mM NaCl, 20 mM HEPES pH 7.4, 1% Triton, 1 mM EDTA). 1 μg of each antibody was conjugated to 25 μL of Protein G Dynabeads for 15 min at room temperature, then washed with TBST. IP was carried out for 1 h at room temperature on a rotating wheel with protein lysates diluted by TBST. For peptide blocks, antibodies were pre-incubated with peptide, conjugated to Dynabeads for 15 min, and lysate was added for 1 h at room temperature. After IP, samples were washed in extraction buffer, then eluted from the Dynabeads by heating in LDS (Thermo) at 70C for 10 min. Supernatants were then run on Mini TGX gels (Biorad) for western blot with anti-Flag (Sigma) antibody.

### Western blotting

For western blots displayed in Figs. 4 and Additional file 3: Figure S4 the following parameters were used: wet transfer (1% [w/v] SDS / 20% [v/v] methanol in transfer buffer) for 90 min / 70 V / 4 °C, PVDF membrane (0.45 μm), HRP-conjugated secondary antibodies (see below), and chemiluminescent HRP detection (substrate: Millipore Sigma #WBLUF0100). Blocking was done overnight at 4 °C using 5% (w/v) nonfat dry milk in TBST (20 mM Tris-Cl, 137 mM NaCl, 0.1% Tween 20), pH 7.6. Primary antibodies were applied overnight at 4 °C in 5% (w/v) BSA in TBST, pH 7.6. Secondary antibodies were applied for 2 h at room temperature in 5% (w/v) BSA in TBST, pH 7.6. Where appropriate, total protein quantities were estimated using a commercial Bradford reagent. An ImageQuant LAS-4000 system (GE Healthcare) was used for blot imaging on the high sensitivity setting with incremental image capture. ECL signal capture times displayed varied with target from ~ 1–5 min and were free of pixel saturation in any signal displayed in the figures. Anti-ORF1p (Millipore Sigma #MABC1152) was used at 0.4 μg/ml; anti-ORF2p (this study) clone MT5 was used at 0.13 μg/ml and clone MT9 was used at 0.71 μg/ml; anti-GAPDH (Cell Signaling #2118) was used at 0.02 μg/ml. Secondary antibodies: anti-mouse HRP conjugate (GE Lifesciences #NV931) and anti-rabbit HRP conjugate (GE Lifesciences #NV934) were used at 1:10,000. All panels displayed have been ‘auto tone’ calibrated, respectively, in Adobe Photoshop to maximize the visual contrast across the detected signal range.

For western blots displayed in Figs. 2 and 3**,** cells were lysed in RIPA buffer, vortexed, and supernatants quantified by BCA. Lysates were reduced in LDS with beta-mercaptoethanol and then polyacrylamide gel electrophoresis was performed on 4–20% Protean Mini TGX gels (Biorad) and transferred to Immobilon PVDF membranes for 15 min using mini TGX settings on the Trans-Blot-Turbo system (Biorad). Membranes were incubated with primary antibodies overnight at 4 °C (rabbit anti-ORF2 mAbs at 1:1000; mouse anti-Flag M2 (Sigma F1804) at 1:2000), secondary antibodies (all from Licor and used at 1:10,000 dilutions; as appropriate: goat anti-mouse IR680, goat anti-rabbit IR680, goat anti-mouse IR800, goat anti-rabbit IR800) for 1 h at room temperature, and detection was carried out on the Odyssey Scanner (Licor).

### Mass spectrometry

Peptides were resuspended in 10 μL 5% (v/v) methanol, 0.2% (v/v) formic acid and half was loaded onto an EASY-Spray column (Thermo Fisher Scientific, ES800, 15 cm × 75 μm ID, PepMap C18, 3 μm) via an EASY-nLC 1200 interfaced with a Q Exactive Plus mass spectrometer (Thermo Fisher Scientific). Column temperature was set to 35 °C. Using a flow rate of 300 nl/min, peptides were eluted in a gradient of increasing acetonitrile, where Solvent A was 0.1% (v/v) formic acid in water and Solvent B was 0.1% (v/v) formic acid in 95% (v/v) acetonitrile. Peptides were ionized by electrospray at 1.8–2.1 kV as they eluted. The elution gradient length was 10 min for gel bands and 140 min for all gel plugs except, the second set derived from tumor A, where the gradient length was 190 min. Full scans were acquired in profile mode at 70,000 resolution (at 200 m/z). The top 5 (for gel bands) or 25 (for gel plugs) most intense ions in each full scan were fragmented by HCD. Peptides with charge state 1 or unassigned were excluded. Previously sequenced precursors were also excluded, for 4 s (for gel bands) or 30 s (for gel plugs), within a mass tolerance of 10 ppm. Fragmentation spectra were acquired in centroid mode at 17,500 resolution. The AGC target was 2 × 10^5^, with a maximum injection time of 200 msec. The normalized collision energy was 24%, and the isolation window was 2 m/z units.

### Analysis of excised protein bands and candidate phospho-sites

Proteins labeled in Fig. 4a selected for labeling via the following process: The RAW files were converted to MGF format by ProteoWizard [75] and searched against the human protein database with X! Tandem [39], using the following settings: fragment mass error - 10 ppm; parent mass error - 10 ppm; cleavage site - R or K, except when followed by P; maximum missed cleavage sites - 1; maximum valid peptide expectation value - 0.1; fixed modification - carbamidomethylation at C; potential modification - oxidation at M; include reversed sequences - yes. Parameters for the refinement search were: maximum valid expectation value - 0.01; potential modifications - deamidation at N or Q, oxidation or dioxidation at M or W; unanticipated cleavage - yes. For each protein ID list, the proteins were ranked by log E-value; keratins, proteins ranked below trypsin, and non-human proteins were removed; if multiple proteins remained, the nth protein (*n* > 1) was removed if (a) it is homologous to a higher-ranked protein or (b) does not have within 50% the number of PSMs of the top-ranked remaining protein; remaining proteins were listed as IDs for each band. For identification of candidate phosphorylation sites using X! Tandem, the RAW files from tumor IPs (corresponding to Fig. 5) were converted to MGF, these were searched against orthogonalized ORF protein sequences (described below), and included the following additional potential modifications during the refinement search: Phospho@S, Phospho@T, Phospho@Y. The best scoring PSMs for phospho S18 and S27 are displayed in Additional file 6: Table S2. The X! Tandem .xml output files are available via ProteomeXchange with identifier PXD013743.

### Label-free quantitative analysis

Processing RAW data in MaxQuant: we used MaxQuant v1.6.5.0 [76, 77] with default settings and the following adjustments (in brief). Trypsin/P cleavage. Modifications included in protein quantification: Oxidation (M); Acetyl (Protein N-term); Carbamidomethyl (C). Phospho (STY) was searched but excluded from quantification along with unmodified counterpart peptides. Label min. Ratio count: 2. Match between runs (within groups of cognate experiments and controls): True. Second peptides: True. Stabilize large LFQ ratios: True. Separate LFQ in parameter groups: False. Require MS/MS for LFQ comparisons: True. We used a protein database composed of the Uniprot human proteome (reviewed), supplemented with non-redundant ORF1p and ORF2p sequences. To increase detection sensitivity, we orthogonalized our ORF1 loci database (from above, *Detection of L1 ORF peptides in CPTAC data*) within the context of our detected peptides in two steps: (a) retaining loci for which at least one unique peptide was observed and (b) in cases that a peptide was not assigned to any loci in previous step and was commonly shared by several loci, we included only one representative sequence from the group, which was the most different one to consensus ORF1 (L1RE1). The RAW and MaxQuant processed files are available for download via ProteomeXchange with identifier PXD013743.

### Custom post-processing in R: code can be obtained at https://github.com/moghbaie/L1_CRC_IP_MS

#### Data preparation

(a) Remove contaminants and reverse protein entries (provided by MaxQuant) and IGHG1. (b) Log_2_ transformation of LFQ MS intensities. (c) Remove proteins with zero values across all cases and controls in a tissue. (d) Impute small values in scenarios that all replicates had zero values for intensity in either cases or controls: we calculated the average (mean) and standard deviation (std) of non-zero values of each replicate and produced small values with the uniform random function between mean – 2*std. and mean – 3*std. (e) Impute values for proteins that have zero intensities in one or two replicates in either cases or controls: we built a distribution of deltas from replicates with non-zero protein intensities: $delta=\frac{\left({Int}_{rep1}-{Int}_{rep2}\right)\ }{mean\left({Int}_{rep1},{Int}_{rep2}\right)}$; Calculate *μ*_*delta*_ , *sd*_*delta*_; Calculate new delta and new Intensity: ${delta}_{new}= rnorm\left( mu={\mu}_{delta}, sd=\frac{sd_{delta}}{mean(correlations)\ast \sqrt{2}}\right)$

$$ {I}_{new}= mean\left( In{t}_{other}\right)\ast abs\left(1+ delt{a}_{new}\right) $$

#### Variance Analysis

(a) For tumor A, we performed t-tests between anti-ORF1p IPs and IgG-controls. For tumor B and C, we performed t-tests between tumor and normal tissue after anti-ORF1p IP, as well as between tumor anti-ORF1p IPs and IgG-controls. (b) Adjusted *p*-values were calculated using Benjamin-Hochberg method. (c) For each entry log_2_ fold change was calculated between case and control average intensity. (d) Significant proteins from all comparison were integrated (p. adjusted ≤0.05 & log_2_ fold change ≥1). We only accept proteins with (non-imputed) MS intensity values in at least two experimental replicates as candidate true positives. Therefore, proteins that passed ANOVA but were represented by less than two MS-derived intensity values were not considered significant. The results are collated in Additional file 6: Table S2.

## Supplementary information

**Additional file 1: Figure S1.** CLUSTAL Alignments of ORF2 protein sequences. ORF2p protein sequences were obtained from the L1Base database of reference L1Hs sequences. (**A**) Alignment of 146 full-length L1 sequences. (**B**) Alignment of 107 ORF2-intact L1 sequences. In the center tiles, black bars indicate amino acid positions where the L1 in that row differs from the CLUSTAL alignment consensus sequence. The ‘% agreement,’ or identity, at each amino acid position is quantified below the center tiles. On the right, the number of amino acid changes of a particular L1 compared to the immunogen, L1RP, is quantified.

**Additional file 2: Table S1.** Antibody epitope matches for 31 'hot' L1Hs loci as described by Brouha et al, corresponding to Fig. 3e. For each locus, we indicate whether the ORF2p sequence is identical to the antibody epitope ("match") or whether it differs from the epitope. For example, the hot element on ac002980 differs in the MT5 epitope by a single amino acid (D in the antibody-derived epitope and H at the locus). The Brouha BAC clone designation, activity rating, and chromosomal positions are included in the chart.

**Additional file 3: Figure S4.** Co-IP/Western blot. Three different segments of Tumor D were used as starting material for anti-ORF1p affinity isolations (α-ORF1p T1–3), including a mock-capture control using mouse IgG affinity medium with tumor extracts (mIgG T1), and matched normal tissue with anti-ORF1p affinity medium (α-ORF1p N). Co-IP of ORF1p/2p ectopically expressed from pMT302 in HEK-293T_LD_ is provided as a comparative positive control. All co-IPs used 100 mg cells or tissues as input. 100% of the co-IP elutions done using patient tissues were analyzed; in contrast, fractions (labeled) of the co-IP from pMT302 in HEK-293T_LD_ were analyzed. ORF1p yields from Tumor D were comparable to those obtained from 1/5th – 1/10th of a co-IP from pMT302/HEK-293T_LD_. However, while ORF2p signal is clearly detectable in 1/5th and closer to the baseline (but still eminently detectable) in 1/10th of a pMT302/HEK-293T_LD_ co-IP, no ORF2p signal was observed in tumor D co-IPs.

**Additional file 4: Figure S2**. Western blot α-ORF1p titer to detect endogenous ORF1p in clarified cell extracts. The concentration of α-ORF1p used is given along the top; the source of each cell extract is given below that, and each accords to Fig. 2e. The quantity of clarified cell extracts used, in μg total protein, follows below each extract source. **I**: clarified extract used as an input for α-ORF1p affinity capture; **S**: immuno-depleted extracts after incubation with α-ORF1p affinity medium. (**Left blot image**) 1x α-ORF1p concentration - ORF1p signal is observed in with ectopic expression (pMT302) and at just above background in PA-1. α-UPF1 provided as a loading control (NYU1.1B6, 1:1000 [79]). (**Right blot image**) 5x α-ORF1p concentration - ORF1p signal is observed in all cases except HeLa Kyoto. An increase in non-specific signal is also observed elsewhere on the blot. α-PCNA is provided as a loading control (Santa Cruz Biotechnology, Inc. #sc-56; 1:1000).

**Additional file 5: Figure S3**. Coomassie G-250 stained gel plugs used for in-gel digestion followed by MS. A panel is shown for every replicate included in the LFQ-MS analysis. (**A**) Tumor A (Krukenberg Carcinoma, Ovary) was subjected to two independent affinity isolations with different parameters (see **Methods**). Each isolation included three replicates using anti-ORF1p-coupled affinity medium to capture ORF1p from the tumor extracts (Tumor A-1 to A-6), and three replicates using mouse IgG-coupled affinity medium to sample non-specific background from the same extracts (mIgG A Ctrl-1 to Ctrl-6). (**B**) Tumor B (Metastatic Rectal Adenocarcinoma, Liver): including three replicates using anti-ORF1p-coupled affinity medium to capture ORF1p from the tumor extracts (Tumor B-1 to B-3), three replicates using mouse IgG-coupled affinity medium to sample non-specific background from the same extracts (mIgG B Ctrl-1 to Ctrl-6), and three replicates using anti-ORF1p-coupled affinity medium to capture ORF1p from matched normal tissue extracts (Normal B-1 to B-3). (**C**) Tumor C (Adenocarcinoma, Colon): including three replicates using anti-ORF1p-coupled affinity medium to capture ORF1p from the tumor extracts (Tumor C-1 to C-3), three replicates using mouse IgG-coupled affinity medium to sample non-specific background from the same extracts (mIgG C Ctrl-1 to Ctrl-6), and three replicates using anti-ORF1p-coupled affinity medium to capture ORF1p from matched normal tissue extracts (Normal C-1 to C-3).

**Additional file 6: Table S2.** Summary of the MS-based proteomic results, including identified and statistically significant proteins, proteins observed in other studies, ORF1 loci detected, and phospho-S18/S27 PSMs

## Data Availability

Proteomics data. The mass spectrometry proteomics data have been deposited to the ProteomeXchange Consortium via the PRIDE [78] partner repository with the dataset identifier PXD013743. R code. https://github.com/moghbaie/L1_CRC_IP_MS
