# Supplementary material for: LINE-1 ORF2p expression is nearly imperceptible in human cancers
Source: Mob DNA. 2019 Dec 31;11:1. doi: 10.1186/s13100-019-0191-2 (PMC6937734; doi:10.1186/s13100-019-0191-2)
Supplement: Supplementary file 1 — Additional file 1: Figure S1. CLUSTAL Alignments of ORF2 protein sequences. ORF2p protein sequences were obtained from the L1Base database of reference L1Hs sequences. (A) Alignment of 146 full-length L1 sequences. (B) Alignment of 107 ORF2-intact L1 sequences. In the center tiles, black bars indicate amino acid positions where the L1 in that row differs from the CLUSTAL alignment consensus sequence. The ‘% agreement,’ or identity, at each amino acid position is quantified below the center tiles. On the right, the number of amino acid changes of a particular L1 compared to the immunogen, L1RP, is quantified. [file 13100_2019_191_MOESM1_ESM.pdf]

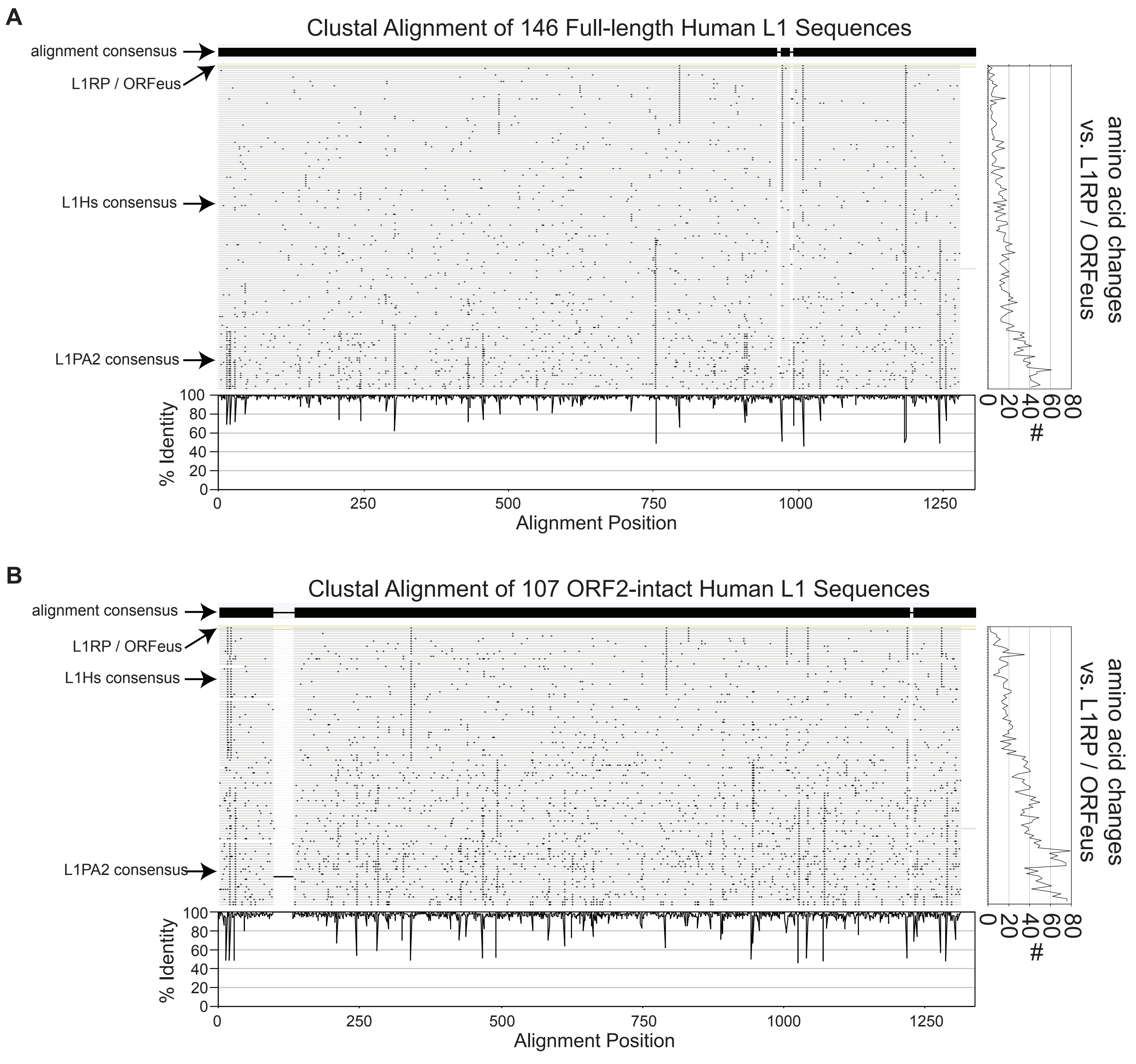
A
Clustal Alignment of 146 Full-length Human L1 Sequences
alignment consensus
L1RP / ORFeus
L1Hs consensus
L1PA2 consensus
amino acid changes vs. L1RP / ORFeus
0 20 40 60 80
#
% Identity
100 80 60 40 20 0
0 250 500 750 1000 1250
Alignment Position
B
Clustal Alignment of 107 ORF2-intact Human L1 Sequences
alignment consensus
L1RP / ORFeus
L1Hs consensus
L1PA2 consensus
amino acid changes vs. L1RP / ORFeus
0 20 40 60 80
#
% Identity
100 80 60 40 20 0
0 250 500 750 1000 1250
Alignment Position
