# Supplementary material for: LINE-1 ORF2p expression is nearly imperceptible in human cancers
Source: Mob DNA. 2019 Dec 31;11:1. doi: 10.1186/s13100-019-0191-2 (PMC6937734; doi:10.1186/s13100-019-0191-2)
Supplement: Supplementary file 3 — Additional file 3: Figure S4. Co-IP/Western blot. Three different segments of Tumor D were used as starting material for anti-ORF1p affinity isolations (α-ORF1p T1–3), including a mock-capture control using mouse IgG affinity medium with tumor extracts (mIgG T1), and matched normal tissue with anti-ORF1p affinity medium (α-ORF1p N). Co-IP of ORF1p/2p ectopically expressed from pMT302 in HEK-293TLD is provided as a comparative positive control. All co-IPs used 100 mg cells or tissues as input. 100% of the co-IP elutions done using patient tissues were analyzed; in contrast, fractions (labeled) of the co-IP from pMT302 in HEK-293TLD were analyzed. ORF1p yields from Tumor D were comparable to those obtained from 1/5th – 1/10th of a co-IP from pMT302/HEK-293TLD. However, while ORF2p signal is clearly detectable in 1/5th and closer to the baseline (but still eminently detectable) in 1/10th of a pMT302/HEK-293TLD co-IP, no ORF2p signal was observed in tumor D co-IPs. [file 13100_2019_191_MOESM3_ESM.pdf]

Figure S4

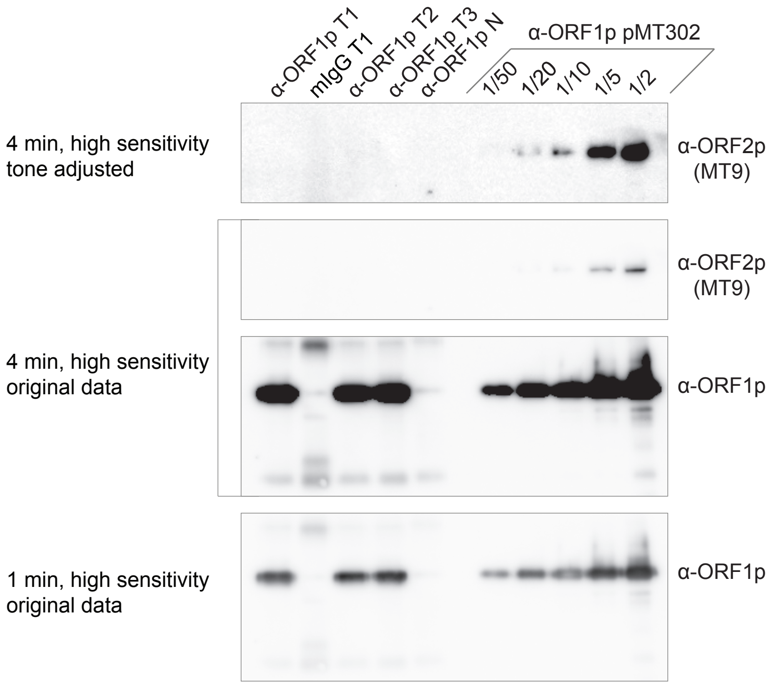

Tumor D: Metastatic sigmoid colon cancer, Liver
Three different metastatic segments were compared: T1, T2, T3
