# Supplementary material for: LINE-1 ORF2p expression is nearly imperceptible in human cancers
Source: Mob DNA. 2019 Dec 31;11:1. doi: 10.1186/s13100-019-0191-2 (PMC6937734; doi:10.1186/s13100-019-0191-2)
Supplement: Supplementary file 4 — Additional file 4: Figure S2. Western blot α-ORF1p titer to detect endogenous ORF1p in clarified cell extracts. The concentration of α-ORF1p used is given along the top; the source of each cell extract is given below that, and each accords to Fig. 2e. The quantity of clarified cell extracts used, in μg total protein, follows below each extract source. I: clarified extract used as an input for α-ORF1p affinity capture; S: immuno-depleted extracts after incubation with α-ORF1p affinity medium. (Left blot image) 1x α-ORF1p concentration - ORF1p signal is observed in with ectopic expression (pMT302) and at just above background in PA-1. α-UPF1 provided as a loading control (NYU1.1B6, 1:1000 [79]). (Right blot image) 5x α-ORF1p concentration - ORF1p signal is observed in all cases except HeLa Kyoto. An increase in non-specific signal is also observed elsewhere on the blot. α-PCNA is provided as a loading control (Santa Cruz Biotechnology, Inc. #sc-56; 1:1000). [file 13100_2019_191_MOESM4_ESM.pdf]

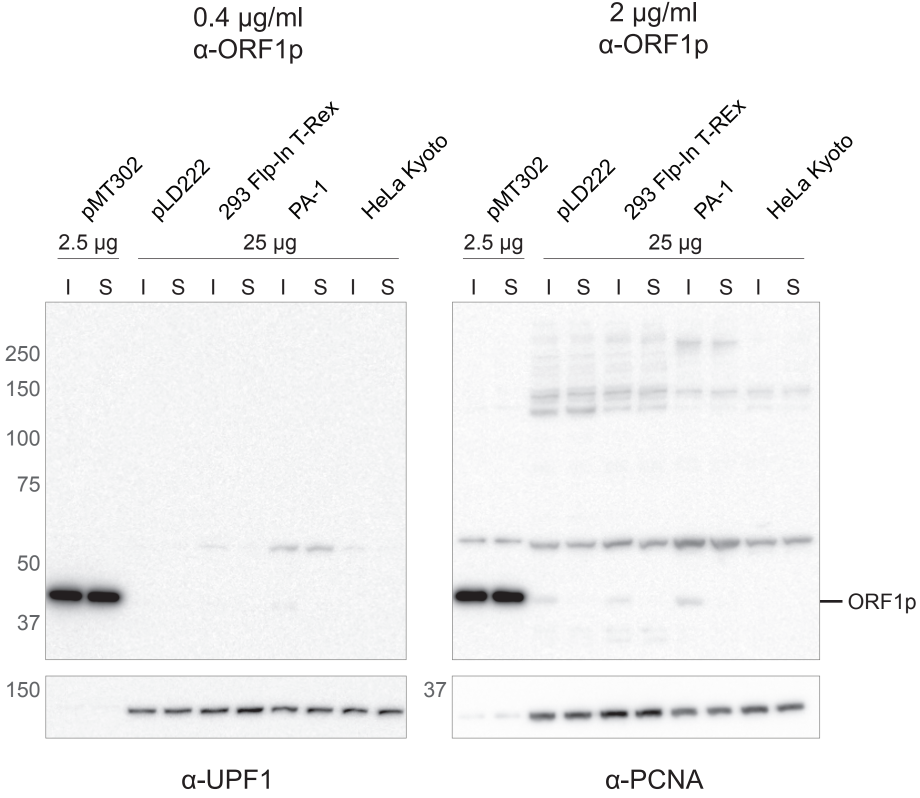
0.4 µg/ml
α-ORF1p
2 µg/ml
α-ORF1p
pMT302
pLD222
293 Flp-In T-Rex
PA-1
HeLa Kyoto
pMT302
pLD222
293 Flp-In T-REx
PA-1
HeLa Kyoto
2.5 µg
25 µg
2.5 µg
25 µg
I S I S I S I S I S
I S I S I S I S I S
250
150
100
75
50
37
150
37
ORF1p
α-UPF1
α-PCNA
