# Supplementary figures and images for: LINE-1 ORF2p expression is nearly imperceptible in human cancers
Source: Mob DNA. 2019 Dec 31;11:1. doi: 10.1186/s13100-019-0191-2 (PMC6937734; doi:10.1186/s13100-019-0191-2)

**A.**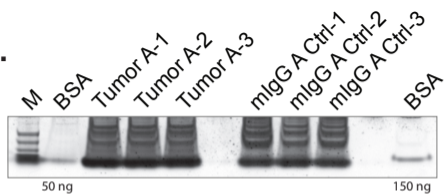**B.**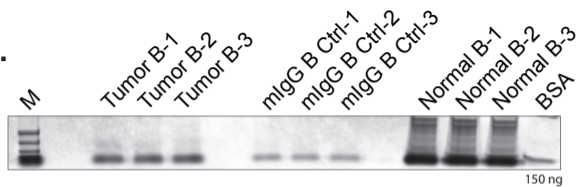**C.**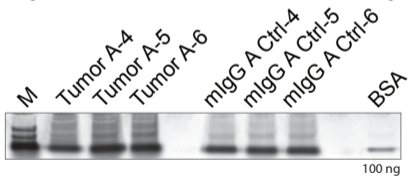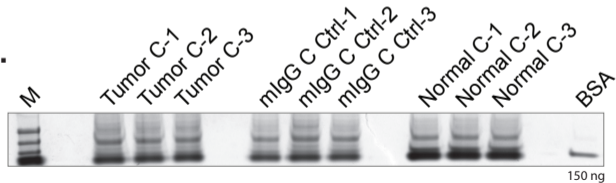

Supplement: Supplementary file 5 — Additional file 5: Figure S3. Coomassie G-250 stained gel plugs used for in-gel digestion followed by MS. A panel is shown for every replicate included in the LFQ-MS analysis. (A) Tumor A (Krukenberg Carcinoma, Ovary) was subjected to two independent affinity isolations with different parameters (see Methods). Each isolation included three replicates using anti-ORF1p-coupled affinity medium to capture ORF1p from the tumor extracts (Tumor A-1 to A-6), and three replicates using mouse IgG-coupled affinity medium to sample non-specific background from the same extracts (mIgG A Ctrl-1 to Ctrl-6). (B) Tumor B (Metastatic Rectal Adenocarcinoma, Liver): including three replicates using anti-ORF1p-coupled affinity medium to capture ORF1p from the tumor extracts (Tumor B-1 to B-3), three replicates using mouse IgG-coupled affinity medium to sample non-specific background from the same extracts (mIgG B Ctrl-1 to Ctrl-6), and three replicates using anti-ORF1p-coupled affinity medium to capture ORF1p from matched normal tissue extracts (Normal B-1 to B-3). (C) Tumor C (Adenocarcinoma, Colon): including three replicates using anti-ORF1p-coupled affinity medium to capture ORF1p from the tumor extracts (Tumor C-1 to C-3), three replicates using mouse IgG-coupled affinity medium to sample non-specific background from the same extracts (mIgG C Ctrl-1 to Ctrl-6), and three replicates using anti-ORF1p-coupled affinity medium to capture ORF1p from matched normal tissue extracts (Normal C-1 to C-3). [file 13100_2019_191_MOESM5_ESM.pdf]
